# Identifying Community-Built Environment’s Effect on Physical Activity and Depressive Symptoms Trajectories Among Middle-aged and Older Adults: Chinese National Longitudinal Study

**DOI:** 10.2196/64564

**Published:** 2025-01-13

**Authors:** Kaili Zhang, Bowen Huang, Prasanna Divigalpitiya

**Affiliations:** 1 Graduate School of Human-Environment Studies Kyushu University Fukuoka Japan; 2 Zigong Academy of Urban Planning and Design Zigong, Sichuan Province China; 3 Faculty of Human-Environment Studies Kyushu University Fukuoka Japan

**Keywords:** community-built environment, physical activity, depressive symptom trajectories, middle-aged and older adults, latent growth curve modeling, longitudinal study

## Abstract

**Background:**

The effects of physical activity (PA) across different domains and intensities on depressive symptoms remain inconclusive. Incorporating the community-built environment (CBE) into longitudinal analyses of PA’s impact on depressive symptoms is crucial.

**Objective:**

This study aims to examine the effects of PA at different intensities—low-intensity PA (eg, walking activities) and moderate-to-vigorous-intensity PA (eg, activities requiring substantial effort and causing faster breathing or shortness of breath)—across leisure-time and occupational domains on depressive symptom trajectories among middle-aged and older adults. Additionally, it investigated how CBEs influence depressive symptoms and PA trajectories.

**Methods:**

This longitudinal study included 6865 middle-aged and older adults from the China Health and Retirement Longitudinal Survey. A CBE variable system was developed using a community questionnaire to assess attributes of the physical built environment. Depressive symptoms were measured using the Center for Epidemiologic Studies Depression Scale. Latent growth curve modeling was applied to analyze 3 waves of the cohort data (2015, 2018, and 2020) to explore the differential effects of PA on depressive symptoms and the role of the CBE.

**Results:**

In the 2015 and 2018 waves, higher low-intensity leisure-time physical activity (LTPA) was associated with lower depressive symptoms (β=–.025, *P*=.01 and β=–.027, *P*=.005, respectively). Across all waves, moderate-to-vigorous-intensity LTPA showed no significant predictive effects (*P*=.21 in 2015, *P*=.57 in 2018, and *P*=.85 in 2020, respectively). However, higher occupational physical activity (OPA), particularly at moderate-to-vigorous intensities, was consistently associated with higher depressive symptoms. Parallel process latent growth curve modeling revealed that the initial level of total LTPA negatively predicted the initial level of depressive symptoms (β=–.076, *P*=.01). OPA exhibited dual effects, positively predicting the initial level of depressive symptoms (β=.108, *P*<.001) but negatively predicting their upward trajectory (β=–.136, *P*=.009). Among CBE variables, better infrastructure conditions (β=–.082, *P*<.001) and greater accessibility to public facilities (β=–.036, *P*=.045) negatively predicted the initial level of depressive symptoms. However, greater accessibility to public facilities positively predicted the upward trajectory of depressive symptoms (β=.083, *P*=.04). Better infrastructure conditions (β=.100, *P*=.002) and greater accessibility to public transport (β=.060, *P*=.01) positively predicted the initial level of total LTPA. Meanwhile, better infrastructure conditions (β=–.281, *P*<.001) and greater accessibility to public facilities (β=–.073, *P*<.001) negatively predicted the initial level of total OPA. Better infrastructure conditions positively predicted the declining trajectory of total OPA (β=.100, *P*=.004).

**Conclusions:**

This study underscores the importance of considering the differential effects of PA across domains and intensities on depressive symptoms in public policies and guidelines. Given the influence of the environment on PA and depressive symptoms, targeted community measures should be implemented.

## Introduction

In communities, the physical built environment and health behaviors, such as physical activity (PA), can significantly influence residents’ mental health [1]. Overall, well-designed community-built environments (CBEs)—including factors such as walkability, appropriate density, and safety—are beneficial in reducing depressive symptoms. By contrast, unfavorable built environments, such as poor-quality housing and insufficient green spaces, may exacerbate depressive symptoms [2]. Exercise and PA have been shown to be as effective as some antidepressant medications in improving depressive symptoms [3].

With the acceleration of the global aging process [4], depression among middle-aged and older adults has become a significant public health issue worldwide. The World Health Organization (WHO) estimates that the prevalence of depression in the global aging population ranges from 10% to 20% [5]. In China, the pooled prevalence of depressive symptoms among seniors aged 60 years and older is approximately 22.7% [6]. Depression poses a serious threat to the mental health of middle-aged and older adults and can also lead to physical illness and an increased risk of death [7].

In all studies examining factors influencing depressive symptoms, PA has become a focal point. PA can be easily incorporated into the daily context of a community, whether in a living or work environment, making it a universally accessible and straightforward intervention. Its practicality and ease of implementation have made PA a key topic in discussions about improving mental health [8]. The beneficial effects of PA on alleviating depressive symptoms are well-documented. Physically inactive individuals are more likely to experience depressive symptoms compared with those who engage in regular PA [9]. Recent studies have shown that this effect is not influenced by age [10]. Middle-aged and older adults are particularly susceptible to depressive disorders due to factors such as the gradual decline in physical function, an increase in chronic illnesses, and the potential for significant life changes, including widowhood, retirement, and living alone after middle age [11]. Older adults who engage in insufficient PA are at a higher risk of depression compared with those who maintain adequate levels of PA [12].

Many studies exploring the relationship between PA and depressive symptoms have primarily focused on exercise-based PA [13]. However, PA can also occur in occupational settings or outside structured exercise programs. PA shows different health outcomes depending on the context, such as work versus leisure settings [14]. Recently, researchers have begun to examine the “PA paradox”—the concept that leisure-time physical activity (LTPA), such as sports and recreational activities, has positive effects on health, while PA in occupational settings may have negative consequences for both physical and mental health [15]. LTPA, regardless of intensity, is associated with a lower risk of depressive symptoms. By contrast, occupational PA (OPA) or transportation-related PA does not show a similar reduction in the risk of depressive symptoms [16]. Workers who engage in work-related physical activities tend to report higher levels of depressive symptoms [17], and individuals with the highest levels of OPA are more likely to experience depressive symptoms [18]. Globally, a significant proportion of people continue to engage in manual labor, particularly in many developing countries. Therefore, it is important to distinguish between different domains or motivations when examining the effect of PA on depressive symptoms, as this distinction may lead to entirely different results. However, only a small number of studies have made such distinctions to examine the effects of various PA domains on depressive symptoms [19].

There are still several limitations and gaps in the existing research on the effects of PA on depressive symptoms. First, while the beneficial effects of exercise on depressive symptoms are well-established, the complex impacts of different domains (such as exercise and work) or PA intensity remain inconclusive [20]. There is still a lack of basic evidence, particularly for middle-aged and older adults, and further research is urgently needed to provide clearer insights. Second, most studies on the effect of PA on depressive symptoms offer only a cross-sectional perspective, with few utilizing cohort data to explore the longitudinal impact. By focusing on individual trajectories and patterns over time, longitudinal studies can provide valuable insights into the developmental continuum, help determine the stability of the association between PA and depressive symptoms, and analyze the dynamics of these interactions [21].

Furthermore, the CBE is an important factor influencing mental health, including depressive symptoms and mental health inequalities [22]. Depressive symptoms are associated with the built environment, and different physical attributes of the community may impact them through various behavioral or social pathways [23]. Many characteristics of the CBE may expose residents to environmental stressors—such as poor housing quality, uncomfortable population densities, low safety, and poor accessibility to amenities—that can negatively affect their mental health [24]. As for built environment measures, the most common ones are related to the “5D” elements: density, diversity, design, destination accessibility, and distance to transit [25]. These elements are often evaluated individually or together.

Despite the growing body of literature on the effects of CBE on depressive symptoms, there is still limited evidence concerning middle-aged and older adults. Few studies have highlighted that improving community walkability, particularly the accessibility and safety of public transportation, is crucial for middle-aged individuals with depression [2]. Higher levels of residential greenery have been significantly associated with lower odds of depressive symptoms among older adults living in the community [26]. Additionally, high population density and proximity to destinations contribute to better mental health, while walkable environments are linked to reduced depression, particularly among Asian women [27]. Conversely, the availability of local retail stores has been associated with an increased likelihood of depressive symptoms in older men [28]. Thus, inconsistent results may arise in different contexts or regions. Therefore, it is crucial to consider the local community context when assessing the effects of CBE on depressive symptoms and PA. Furthermore, among longitudinal studies exploring the relationship between PA and depressive symptoms, few have taken CBE into account [29]. Therefore, providing insights into how CBE influences the trajectories of depressive symptoms and PA is invaluable.

To address these gaps, this study focused on middle-aged and older adults aged 45 years and older in China, using data from 3 waves of repeated measurements to identify the trajectory of depressive symptoms, various types of PA, and the built environment of communities. The study aimed to answer the following 2 research questions: (1) What are the longitudinal effects of LTPA and OPA on depressive symptoms among middle-aged and older adults? (2) How does the CBE influence the trajectory of depressive symptoms and the 2 domains of PA in this population?

## Methods

### Data Source and Study Sample

The data for this study were obtained from the latest 3 waves of tracking data (2015, 2018, and 2020) from the China Health and Retirement Longitudinal Survey (CHARLS), a large microsurvey database. CHARLS covers 450 communities across 28 provinces in China, using a multistage stratified probability proportional to size sampling method to conduct the survey. A detailed description can be found in Appendix S1 in Multimedia Appendix 1 (see also [30-32]). As part of the International Survey of Health in Aging, CHARLS has been extensively utilized in interdisciplinary studies focusing on aging and health issues [33].

Figure S1 in Multimedia Appendix 1 illustrates the sample screening process. For each wave of data, this study excluded samples with missing PA data (missing PA data were randomly absent: participants were randomly selected to answer PA-related questions, and the absence of available records indicated that they did not respond to PA-related questions) and missing depressive symptom scores (individuals who left more than 2 questions unanswered on the scale were considered invalid for the depressive symptom measure. Among the respondents excluded from the analysis, approximately 80% had missing data for all 10 items on the scale in each wave). Additionally, samples lost due to attrition or participant death were excluded. After matching data across the 3 waves, participants younger than 45 years were also excluded. This resulted in a final sample of 6865 respondents, with 20,595 observation points. For samples with missing values for sex and education level, data from adjacent waves were used.

### Ethics Approval

The original data collection was approved by the Biomedical Ethics Review Committee of Peking University (IRB00001052-11015). All data used in this study were fully anonymized, and participants provided informed consent. The data for this study were registered and obtained through the official CHARLS website [34].

### Depressive Symptoms

Depressive symptoms were assessed using the Center for Epidemiologic Studies Depression Scale (CES-D), as provided by CHARLS. Respondents answered 10 questions reflecting feelings and behaviors associated with depression [35]. The total score, which ranged from 0 to 30, was calculated by summing the responses to these 10 questions, with reverse scoring applied to the 2 positive questions. Higher CES-D scores indicated more severe depressive symptoms. The Cronbach α coefficients for the CES-D scale in this study were 0.794, 0.800, and 0.796 in 2015, 2018, and 2020, respectively. In the descriptive statistical analysis, a CES-D score of ≥10 was classified as indicative of depression [36]. Additionally, we calculated the Reliable Change Index (RCI) for the CES-D scores across waves to assess whether changes in participants’ depressive symptom scores over multiple waves were statistically significant (|RCI|>1.96) [37].

### Physical Activity Level

The CHARLS used items similar to those in the International Physical Activity Questionnaire (short form) to assess respondents’ levels of PA. It collected data on the duration and weekly frequency of each type of PA: low intensity (walking activities), moderate intensity (activities that cause faster breathing than usual, such as cycling, playing table tennis, and practicing yoga), and high intensity (activities that cause shortness of breath, such as fast running and playing basketball) [38]. This study used the metabolic equivalent (MET) method to calculate PA levels. To determine an individual’s weekly MET for each PA intensity, we multiplied the corresponding duration, frequency, and coefficient for each activity. Following previous studies [30,31], we performed data organization and calculations for the 3 intensity levels of PA, including data cleaning, outlier elimination, and data truncation. Details regarding the CHARLS Questionnaire on PA, as well as the data processing and calculation methods used in this study, are provided in Appendix S1 in Multimedia Appendix 1. We scaled all PA data by a factor of 100 to meet the data requirements for statistical analysis before inputting them into the model calculation. As a result, the actual unit of MET reported in the models was 100 MET minutes per week.

The CHARLS database collected information on the purpose of each PA (4 categories: work, exercise, recreation, and others), allowing for the examination of the differential effects of various PA domains on depressive symptoms. Activities labeled as “other” in CHARLS were not explicitly defined and included a mix of multiple purposes, so they were excluded from the calculations for this study. PA data were categorized into 2 domains: LTPA and OPA. Within each domain, PA was further classified by intensity: low intensity (walking activities) and moderate-to-vigorous intensity (activities that require significant effort and lead to faster breathing or shortness of breath). The corresponding MET values were calculated and aggregated.

### Construction of the Community-Built Environment Variables System

In this study, a set of variables was developed to assess the physical built environments of communities based on the community questionnaire data set provided by CHARLS. This data set was released in a single issue as part of the national baseline survey, updated in December 2013, with no subsequent updates in later waves. Based on the concepts of 3D, 5D, and other typical indicator systems and their extensions [25,39], and considering data availability and the characteristics of middle-aged and older adults, this study selected 7 core variables to evaluate the CBE attributes of each community: residential density, density of public facilities, diversity of sports venues, diversity of senior care facilities, accessibility of public facilities, accessibility of public transport, and infrastructure conditions. These variables are outlined in Table 1.

**Table 1:** Community-built environment variable system constructed from the community questionnaire of the China Health and Retirement Longitudinal Survey.

Built environment element	Variable	Calculation method: unit (weight)
Density	Residential density Density of public facilities	Community population/total area of the community: pieces/km2 (N/A^a^) The total number of basic public facilities in the community/total area of the community: pieces/km2 (N/A)
Diversity	Diversity of sports venues Diversity of senior care facilities	Whether or not the community has any of the 8 sports venues: a “yes” answer will be scored as 1 point for that venue, a “no” answer will be scored as 0 points for that venue, and the total values will be added up. The maximum score is 8: points (N/A) Whether or not the community has any of the 7 senior care facilities; diversity levels were calculated as described above.
Destination accessibility	Accessibility of public facilities	The average distance from the community office to the most commonly used type of each facility (if this facility is located within the community, the answer is 0): km (N/A).
Distance to transit	Accessibility of public transport	The actual distance from the community office to the most commonly used bus stop: km (43.86%). The total number of actual bus lines accessible to the community: pieces (56.14%).
Infrastructure	Infrastructure conditions	Pavement quality of the main roads, where pathway/dirt/unpaved road=1 point, sandstone road=2 points, and paved road=3 points: points (13.62%) The total number of days roads are impassable: days (12.31%) The proportion of households that use purified tap water: percentage (18.30%) Availability of sewer system in the community. yes=1 point and no=0 points: points (17.56%) The level of waste disposal, where moved away by truck=5 points, buried in the village=4 points, burn away=3 points, put into nearby river=2 points, and do not manage=1 point: points (17.13%) The proportion of households that use electricity: percentage (17.07%) The condition of community toilets, where inside toilet with water=5, inside toilet without water=4, outside toilet with water=3, outside public toilet without water=2, and open air=1: points (4.00%)

^a^N/A: not applicable.

Among the variables, the accessibility of public transport and infrastructure conditions were assessed by calculating multiple subvariables. The CRITIC weighting method was used to determine the weight of each subvariable. Detailed information on the content of the facilities and the source of the question numbers can be found in Table S1 in Multimedia Appendix 1. Furthermore, due to limitations in the survey data, this study could not include variables such as green spaces and road network connectivity, despite their recognized importance [40,41]. All CBE variables were normalized using the min-max normalization method, resulting in a processed data range of 0-1, before being included in the model calculations. Further details can be found in Appendix S1 in Multimedia Appendix 1.

### Covariates

Three types of variables were used as control variables: demographic and socioeconomic status, health behavior and status, and housing characteristics. These variables were selected due to their association with mental health levels or their potential impact on depressive symptoms [42]. Baseline data were chosen as covariates for inclusion in the model calculations. Specifically, demographic and socioeconomic status included age, sex (male or female), education level (categorized into 4 groups: illiteracy; primary and lower; middle, high, and vocational school; and 3-year college/bachelor’s degree and higher), marital status (married or other), cohabiting status (with or without a cohabiting partner), and personal annual income. Personal annual income encompasses salaries and transfer income, such as pensions, benefits, and subsidies.

Health behavior and status included smoking (current smoker or nonsmoker), drinking (categorized into 3 groups: drinking more than once a month, drinking less than once a month, and nondrinker), the number of chronic diseases (total of 10, as outlined in Appendix S2 and Table S2 in Multimedia Appendix 1), and disability status (disabled or nondisabled). Housing characteristics were assessed based on the presence of various amenities: elevator, barrier-free facilities, toilet flushing, electricity, running water, bathing facility, gas/natural gas, heating, broadband, air purifier, and tidiness, for a total of 11 items. One point was assigned for each present characteristic or amenity, and the total score was calculated. Higher scores indicate better housing quality.

### Statistical Analysis

This study utilized latent growth curve modeling (LGCM) to analyze the trajectories and longitudinal associations among the variables. LGCM, a variant of structural equation modeling, is a statistical method commonly used in longitudinal studies with latent variables [43]. It uses the intercept to represent the initial level and the slope to capture the rate of change. First, an unconditional linear LGCM was applied to each of the 3 measures of PA and depressive symptoms to examine their trajectories in middle-aged and older adults. In the second step, all control variables were incorporated into a linear growth model to construct a conditional linear LGCM for depressive symptoms. PA was included as a time-varying covariate to examine the effect of each wave of PA on depressive symptoms. To test whether these effects remained stable over time (2015-2018-2020), equality constraints were applied to the effects at each wave, and likelihood ratio tests (LRTs) were conducted to assess the statistical equivalence of these effects. In the third step, an LGCM was constructed for both depressive symptoms and PA, with 7 categories of built environment (CBE) as time-invariant covariates. A parallel process LGCM was used to examine the dynamic associations among CBE, PA, and depressive symptoms. The study also incorporated the adjusted individual weights provided by CHARLS for each wave of data in all models to minimize representational bias due to response differences. The acceptable thresholds for model fit indices were as follows: Comparative Fit Index>0.90, Tucker-Lewis Index>0.90, root mean square error of approximation<0.08, and standardized root mean square residual<0.08 [44]. Additionally, the Harman single-factor test was conducted to examine the data for common method bias. The total variance explained by the first common factor was 19.76% (<40%), indicating that the study was not significantly affected by common method bias [45]. Data cleaning was performed using Stata 18.0 (StataCorp), all LGCM analyses were conducted with Mplus 8.10 (Muthén and Muthén), and the Harman single-factor test was executed using IBM SPSS 26.

## Results

### Characteristics of the Sample at Baseline

A total of 6865 participants were included in the study sample, with the detailed statistical characteristics presented in Table S2 in Multimedia Appendix 1. The male-to-female ratio in the sample was 0.88, indicating a nearly balanced distribution. The respondents’ education levels were relatively low: of the 6865 participants, 4652 (67.76%) did not receive education beyond secondary school, reflecting the social context of the middle-aged and older adult generation in China. The marriage rate in the sample was very high (6836/6865, 99.58%), and a majority of respondents (5767/6865, 84%) lived with a cohabiting partner. The mean annual personal income of participants was 15,712.63 Chinese yuan (1 yuan=US $0.14). Among the respondents, the majority were nonsmokers (4986/6865, 72.63%) and nondrinkers (4400/6865, 64.09%). On average, participants reported having 2.10 chronic diseases, and the average score for housing characteristics was 4.06. Additionally, 872 (12.70%) participants reported having a disability.

### Developmental Trajectories of Physical Activity and Depressive Symptoms

The statistics for depressive symptoms and various types of PA among middle-aged and older adults across the 3 data waves are presented in Table 2. The proportion of participants with depression (CES-D scores≥10) ranged from approximately 30% to 40% over the study period (2178/6865, 31.73%, in 2015; 2469/6865, 35.97%, in 2018; and 2666/6865, 38.83%, in 2020). Additionally, in each wave, participants consistently reported higher levels of OPA than LTPA. The amount of moderate-to-vigorous-intensity OPA was greater than that of low-intensity OPA, while low-intensity LTPA was more prevalent than moderate-to-vigorous-intensity LTPA.

Unconditional LGCM was used to examine the developmental trends of each variable. The model fit indices, along with the mean intercepts and slopes for the 3 models, are presented in Table 3. Overall, the models demonstrated a good fit. Depressive symptoms and LTPA levels showed a linear upward trend across the 3 measurement waves (with positive and significant slopes), while OPA levels displayed a linear downward trend. Furthermore, based on the RCI statistics for changes in depressive symptoms (Appendix S2 and Table S3 in Multimedia Appendix 1), despite the overall increasing trend in depressive symptoms, most participants did not show significant changes across waves (–1.96≤RCI≤1.96). Specifically, 5506 of 6865 (80.20%) individuals from 2015 to 2018, 5483 of 6865 (79.87%) individuals from 2018 to 2020, and 5391 of 6865 (78.53%) individuals from 2015 to 2020 showed no significant changes (–1.96≤RCI≤1.96). Over the longer period from 2015 to 2020, 948 of 6865 (13.81%) participants experienced a significant increase (RCI>1.96) in depressive symptoms, while 526 of 6865 (7.66%) participants experienced a significant decrease (RCI<–1.96).

**Table 2:** Statistical characteristics of depressive symptoms and physical activity among middle-aged and older adults across three waves of data.^a^

Variable		Wave 2015	Wave 2018	Wave 2020
**Depressive symptoms, mean (SD)**		7.81 (6.27)	8.46 (6.32)	8.85 (6.46)
	Number of depression, n/N (%)	2178/6865 (31.73)	2469/6865 (35.97)	2666/6865 (38.83)
	Number of nondepression, n/N (%)	4687/6865 (68.27)	4396/6865 (64.03)	4199/6865 (61.17)
**Total LTPA^b^, mean (SD)**		974.38 (2130.84)	1248.28 (2412.47)	1590.66 (2748.37)
	Low-intensity LTPA, mean (SD)	593.47 (1198.14)	792.45 (1356.73)	902.70 (1356.9)
	Moderate-to-vigorous-intensity LTPA, mean (SD)	380.91 (1514.96)	455.83 (1719.31)	687.96 (2140.36)
	Total OPA^c^, mean (SD)	5529.50 (6988.45)	4834.27 (6593.79)	3745.39 (5744.90)
	Low-intensity OPA, mean (SD)	1234.15 (1907.24)	1190.91 (1925.46)	719.76 (1570.59)
	Moderate-to-vigorous-intensity OPA, mean (SD)	4295.35 (5985.89)	3643.36 (5600.78)	3025.63 (5064.63)

^a^A score of the Center for Epidemiologic Studies Depression Scale ≥10 was defined as depression. The unit of physical activity in this table is metabolic equivalent minutes/week.

^b^LTPA: leisure-time physical activity.

^c^OPA: occupational physical activity.

**Table 3:** Estimates and fit index of unconditional LGCMs.^a^

Estimates		LGCM^b^ for depressive symptoms		LGCM for total LTPA^c^			LGCM for total OPA^d^	
		Estimate	SE	Estimate	SE	Estimate		SE
**Means**								
	Intercept	7.855^e^	0.073	9.661^e^	0.246	56.000^e^		0.803
	Slope	0.516^e^	0.038	3.048^e^	0.190	–9.087^e^		0.439
Covariance (intercept with slope)		–0.319	0.486	–18.383^f^	8.409	–46.701^e^		5.570
**Fit index**								
	Chi-square (*df*)	4.180 (1)	N/A^g^	1.182 (1)	N/A	3.442 (1)		N/A
	Root mean square error of approximation (90% CI)	0.022 (0.004-0.045)	N/A	0.005 (0.000-0.033)	N/A	0.031 (0.013-0.053)		N/A
	Comparative Fit Index	0.999	N/A	1.000	N/A	0.998		N/A
	Tucker-Lewis Index	0.998	N/A	0.999	N/A	0.994		N/A
	Standardized root mean square residual	0.006	N/A	0.004	N/A	0.008		N/A

^a^The unit of physical activity in this table is 100 metabolic equivalent minutes/week.

^b^LGCM: latent growth curve modeling.

^c^LTPA: leisure-time physical activity.

^d^OPA: occupational physical activity.

^e^*P*<.001.

^f^*P*<.05.

^g^N/A: not applicable.

### Effects of Leisure-Time and Occupational Physical Activity on Depressive Symptoms

**Figure 1:**
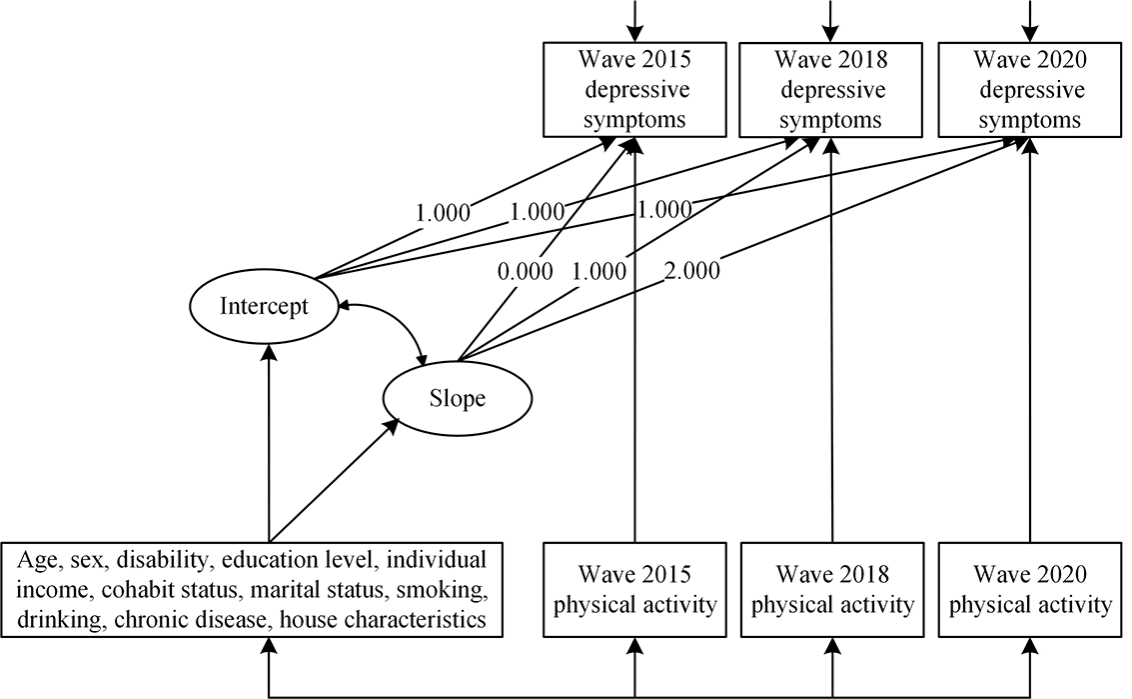
Graphical representation of the time-varying covariate latent growth curve modeling with the depressive symptoms and physical activity as time-varying variables. The 11 covariates as time-invariant covariates were plotted in a box for mapping simplicity. They were calculated concurrently in each latent growth curve model analysis.

Based on the time-varying covariate LGCM specified in Figure 1, the direct effects of all types of PA on depressive symptoms were calculated for each wave, and equality constraints were tested. The results are presented in Table 4. The model fit for all pathways was satisfactory (Appendix S2 and Table S4 in Multimedia Appendix 1). In both 2015 and 2018, total LTPA (β=–.023, *P*=.02 and β=–.025, *P*=.01, respectively) and low-intensity LTPA (β=–.025, *P*=.01 and β=–.027, *P*=.005, respectively) negatively predicted depressive symptoms. However, this effect was not statistically significant in 2020 (*P*=.51). No significant differences were observed in the pathways across multiple waves, as the LRT results were not significant (*P*=.31 in 2015, *P*=.65 in 2018, and *P*=.14 in 2020, respectively). Moderate-to-vigorous-intensity LTPA did not show a significant predictive effect on depressive symptoms in any wave (*P*=.21 in 2015, *P*=.57 in 2018, and *P*=.85 in 2020, respectively).

By contrast, in waves 2015, 2018, and 2020, both total OPA (β=.041, *P*<.001; β=.022, *P*=.02; and β=.027, *P*=.03, respectively) and moderate-to-vigorous-intensity OPA (β=.036, *P*<.001; β=.020, *P*=.04; and β=.019, *P*=.049, respectively) positively predicted depressive symptoms. The positive predictive effect of low-intensity OPA on depressive symptoms was significant only in wave 2015 (β=.030, *P*=.003). Furthermore, the LRT results indicated that the effect of low-intensity OPA on depressive symptoms differed significantly between waves 2015 and 2018 (*P*=.048) and between waves 2015 and 2020 (*P*=.02). Across the 3 waves of data, the effects of both total OPA (*P*=.27 in 2015; *P*=.98 in 2018; and *P*=.33 in 2020, respectively) and moderate-to-vigorous-intensity OPA on depressive symptoms remained stable with no significant differences observed (*P*=.27 in 2015, *P*=.87 in 2018, and *P*=.41 in 2020, respectively). Given the representativeness, statistical significance, and stability of the effects across waves, this study proceeded with the inclusion of total LTPA and total OPA for further analysis.

**Table 4:** Standardized estimate, SE (in parentheses), and significance of the time-varying covariate latent growth curve modeling.^a^

Variables	Wave 2015 depressive symptoms	Wave 2018 depressive symptoms	Wave 2020 depressive symptoms	LRT^b^ 2015 vs 2018	LRT 2018 vs 2020	LRT 2015 vs 2020
2015 Total LTPA^c^	–*0.023 (0.010)^d^*	N/A^e^	N/A	*P*=.31	N/A	N/A
2018 Total LTPA	N/A	–*0.025 (0.010)^d^*	N/A	N/A	*P*=.65	N/A
2020 Total LTPA	N/A	N/A	–0.015 (0.022)	N/A	N/A	*P*=.14
2015 Low-intensity LTPA	–*0.025 (0.010)^d^*	N/A	N/A	*P*=.93	N/A	N/A
2018 Low-intensity LTPA	N/A	–*0.027 (0.010)^f^*	N/A	N/A	*P*=.19	N/A
2020 Low-intensity LTPA	N/A	N/A	–*0.010 (0.010)*	N/A	N/A	*P*=.20
2015 Moderate-to-vigorous-intensity LTPA	–0.012 (0.010)	N/A	N/A	*P*=.18	N/A	N/A
2018 Moderate-to-vigorous-intensity LTPA	N/A	0.005 (0.009)	N/A	N/A	*P*=.57	N/A
2020 Moderate-to-vigorous-intensity LTPA	N/A	N/A	–0.002 (0.009)	N/A	N/A	*P*=.35
2015 Total OPA^g^	*0.041 (0.010)^h^*	N/A	N/A	*P*=.27	N/A	N/A
2018 Total OPA	N/A	*0.022 (0.010)^d^*	N/A	N/A	*P*=.98	N/A
2020 Total OPA	N/A	N/A	*0.027 (0.012)^d^*	N/A	N/A	*P*=.33
2015 Low-intensity OPA	*0.030 (0.010)^f^*	N/A	N/A	**P*=.048^d^*	N/A	N/A
2018 Low-intensity OPA	N/A	0.012 (0.010)	N/A	N/A	*P*=.22	N/A
2020 Low-intensity OPA	N/A	N/A	–0.005 (0.010)	N/A	N/A	**P*=.02^d^*
2015 Moderate-to-vigorous-intensity OPA	*0.036 (0.010)^h^*	N/A	N/A	*P*=.27	N/A	N/A
2018 Moderate-to-vigorous-intensity OPA	N/A	*0.020 (0.010)^d^*	N/A	N/A	*P*=.87	N/A
2020 Moderate-to-vigorous-intensity OPA	N/A	N/A	*0.019 (0.010)^d^*	N/A	N/A	*P*=.41

^a^Italicized values indicate significant results.

^b^LRT: likelihood ratio test.

^c^LTPA: leisure-time physical activity.

^d^*P*<.05.

^e^N/A: not applicable.

^f^*P*<.01.

^g^OPA: occupational physical activity.

^h^*P*<.001.

### Longitudinal Associations Among Community-Built Environment, Physical Activity, and Depressive Symptoms

**Figure 2:**
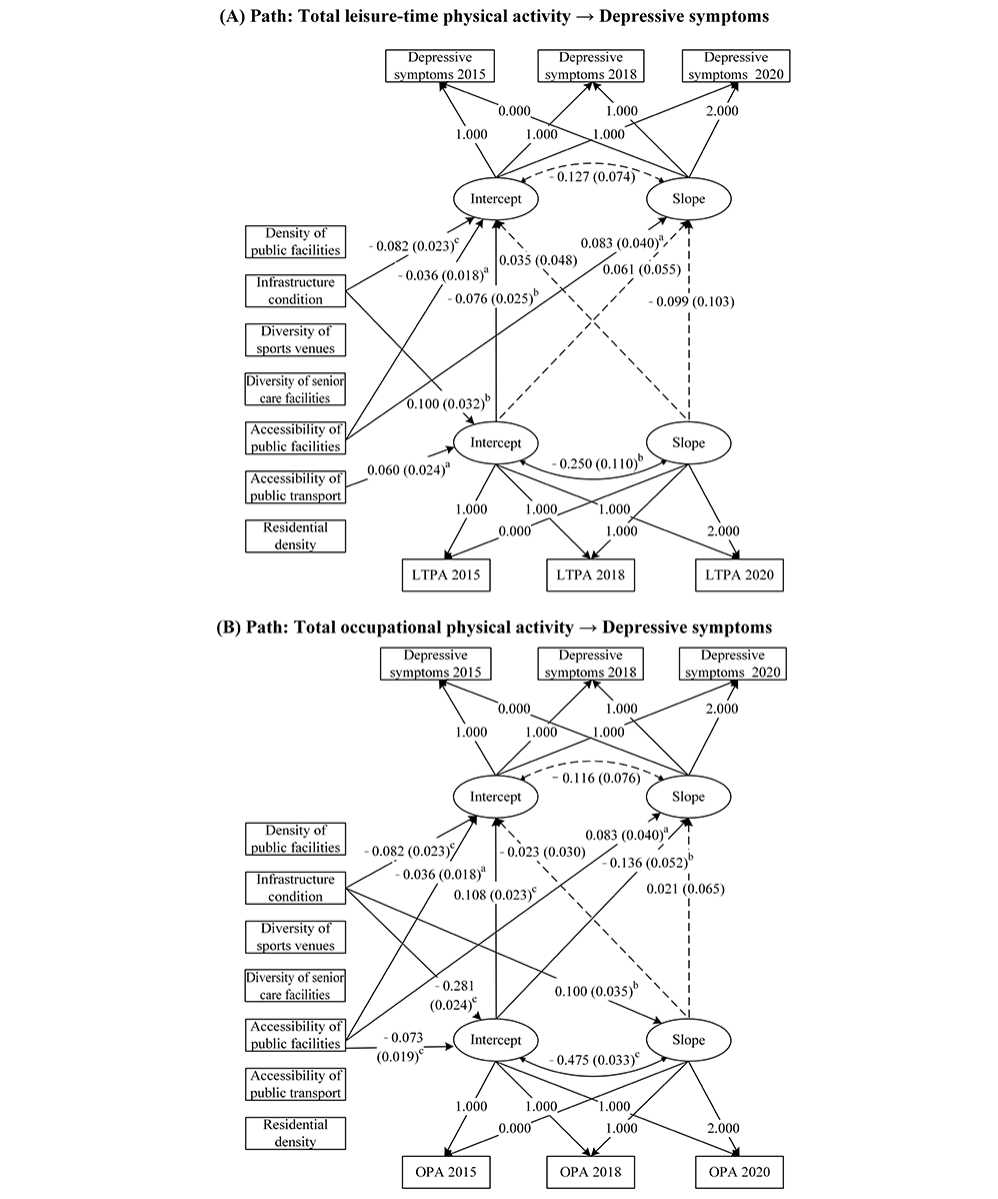
Parallel process latent growth curve modeling to estimate the effect of physical activity on depressive symptoms, with the community-built environment serving as a time-invariant covariate. Dashed lines indicate nonsignificant paths. The nonsignificant paths for the 7 community-built environment variables are not displayed. All control variables are evaluated simultaneously. ^a^*P*<.05; ^b^*P*<.01; ^c^*P*<.001. LTPA: leisure-time physical activity; OPA: occupational physical activity.

This study further used a parallel process LGCM to analyze the dynamic associations between CBE, the 2 domains of PA, and depressive symptoms (Figure 2). The models demonstrate a good fit (Appendix S2 and Table S4 in Multimedia Appendix 1). First, the intercept (initial level) of total LTPA negatively predicted the initial level of depressive symptoms (β=–.076, *P*=.003). The initial level of total OPA positively predicted the initial level of depressive symptoms (β=.108, *P*<.001) and negatively predicted the rate of increase in depressive symptoms (β=–.136, *P*=.009).

Regarding the effect of CBE on the trajectory of depressive symptoms, infrastructure conditions and accessibility of public facilities negatively predicted the initial level of depressive symptoms (β=–.082, *P*<.001 and β=–.036, *P*=.045, respectively). However, accessibility to public facilities positively predicted the rate of increase in depressive symptoms (β=.083, *P*=.04). Additionally, infrastructure conditions (β=.100, *P*=.002) and accessibility of public transport (β=.060, *P*=.01) positively predicted the intercept of total LTPA. By contrast, infrastructure conditions (β=–.281, *P*<.001) and accessibility of public facilities (β=–.073, *P*<.001) negatively predicted the initial level of total OPA. Infrastructure conditions also positively predicted the declining trend of total OPA over time (β=.100, *P*=.004).

## Discussion

### Longitudinal Effect of Physical Activity on Depressive Symptoms Among Middle-Aged and Older Adults

First, the analysis based on the linear unconditional LGCM revealed an upward trend in depressive symptoms among middle-aged and older adults from 2015 to 2020. This finding aligns with previous studies using CHARLS data [33], suggesting that depressive symptoms may be influenced by the aging process. However, further analysis using the RCI indicated that while depressive symptoms showed an upward trend, this change appeared to occur primarily at the group level. At the individual level, fluctuations in depressive symptoms were relatively small for most participants, with only a minority experiencing significant increases. For the majority, changes did not reach statistically significant levels (Table S3 in Multimedia Appendix 1). Additionally, the average levels of depressive symptoms across the 3 waves were slightly below the CES-D clinical screening threshold (Table 2) [36], indicating that most participants exhibited mild or subclinical depressive symptoms. Therefore, the core focus of this study is to understand and explore the potential effects of CBE and PA on changes in depressive symptoms. These changes should not be overly simplified as the onset of clinical depression or an increase in its severity.

Existing studies examining the effects of PA on depressive symptoms often focus on a single domain or intensity level. However, given that PA across different intensities and domains may have varying effects on depressive symptoms [46], our study contributes to the literature by further categorizing PA into low- and moderate-to-high-intensity levels within the domains of LTPA and OPA. These results highlight the importance of the differences observed. By analyzing data from 3 waves of repeated measurements, we found that LTPA, particularly low-intensity LTPA, consistently served as a negative predictor of depressive symptoms among middle-aged and older adults in China. This suggests that greater participation in low-intensity LTPA was associated with fewer depressive symptoms in this population (Table 4). Our findings are consistent with previous studies that emphasize the benefits of moderate and light exercise over vigorous exercise for middle-aged and older adults [47]. Engaging in lower-intensity activities, such as walking, may also help prevent depression [48], with lower LTPA levels being associated with fewer depressive symptoms [20].

However, in all waves of the analysis, we did not observe a significant association between moderate-to-vigorous-intensity LTPA and depressive symptoms. This finding is inconsistent with some existing literature. For example, certain cross-sectional studies have suggested that both low- and moderate-to-vigorous-intensity PA positively impact the mental health of middle-aged and older adults [49]. Furthermore, both small and large amounts of LTPA are associated with a lower prevalence of depression, with comparable effects [19]. These discrepancies may arise from differences in how PA intensity is categorized, the participant selection criteria, or regional characteristics across the studies.

This study also clearly observed that after controlling for socioeconomic factors such as income levels, higher moderate-to-vigorous-intensity OPA was associated with higher depressive symptoms. This negative predictive effect remained significant and consistent across all waves of data (Table 4). This result aligns with previous studies. Research from Brazil and South Korea reported that participants with higher levels of OPA were more likely to experience depressive symptoms [20], and that the higher the level of work-related PA, the greater the likelihood of depressive symptoms [50]. Similarly, OPA appears to be associated with an increased risk of depression [51]. However, these results contradict those of other studies. For instance, research from the United States suggested that OPA does not significantly predict depression regardless of gender [52], while a study from Ghana found that depression decreased as work-related PA increased [53]. As our study controlled for socioeconomic characteristics, these differences suggest that the effect of OPA on depressive symptoms may vary depending on the level of economic development or overall social progress in a given region.

Collectively, these findings support the important perspective that the effects of PA on depressive symptoms vary depending on the domain and intensity of the activity. This study adds to the existing research on the PA paradox and PA parameters [54]. Overall, our findings validate and expand upon the PA paradox in the mental health domain, showing that LTPA, particularly low-intensity exercise in LTPA, is associated with lower depressive symptoms, whereas moderate-to-vigorous-intensity OPA is linked to higher depressive symptoms. These 2 distinct forms of PA exert opposite effects on depressive symptoms in middle-aged and older adults [51]. Leisure exercise and occupational labor, as fundamentally different drivers of PA, likely result in varying degrees of subjective willingness and well-being, which may have divergent long-term effects on mental health [55]. Particularly in many developing countries, moderate-to-vigorous-intensity OPA is often linked to limited individual job skills and is usually accompanied by poorer working conditions and lower levels of welfare protection.

When examining the dynamic effects of PA on depressive symptoms, this study further revealed the heterogeneous characteristics of different domains of PA. Using parallel-processed LGCM, we found that the initial level of total LTPA in middle-aged and older adults negatively predicted the initial level of depressive symptoms. This indicated that individuals with higher initial LTPA levels tended to have lower initial levels of depressive symptoms (Figure 2). This finding is consistent with the results of Heesch et al [56], who reported that low LTPA and walking levels were associated with reduced anxiety and depression scores. One possible explanation is that individuals with depressive symptoms are less likely to engage in PA. Additionally, confounding factors may have influenced this association.

This study also identified, for the first time, that OPA is not only significantly positively associated with the initial level of depressive symptoms, but also significantly predicts the trajectory of change in depressive symptoms. Specifically, middle-aged and older adults with higher initial OPA levels tended to exhibit higher initial levels of depressive symptoms. However, higher OPA levels appeared to suppress the upward trajectory of depressive symptoms over time (Figure 2). This finding highlights the complexity and duality of OPA’s influence on the depressive symptom trajectory. On the one hand, high-intensity or repetitive OPA may increase physical burdens, contributing to higher initial depressive symptoms. On the other hand, the “steeling effect” resulting from prolonged OPA may enhance individuals’ resilience to negative experiences and improve physiological adaptability, thereby mitigating the increase in depressive symptoms over time [57,58]. Moreover, OPA may exert long-term positive effects on mental health through mechanisms such as improving physical metabolism, maintaining a regular daily routine, and reducing sedentary behavior. These benefits may partially offset the negative effects.

### Effects of Community-Built Environment on Trajectories of Depressive Symptoms and Physical Activity

To the best of our knowledge, few studies have attempted to develop indicator systems or variable sets for international microsurvey databases to assess a community’s physical environment. Jones-Smith and Popkin [59] proposed a scale for assessing community contextual characteristics but did not pay enough attention to physical built environments. Taking the CHARLS community questionnaire as an example, this study developed CBE indicators for international microsurvey databases, which can provide references for other studies. Specifically, incorporating the variables of the diversity of senior care facilities and sports venues directly addresses the unique needs of adults aged 45 years or older. Assessing the level of access to senior care and exercise facilities within the community is particularly important for this age group. The density of public facilities and accessibility of public facilities reflect the richness of community services and the convenience of facility distribution, providing valuable insights into middle-aged and older adults’ ease of access to essential daily services. These factors are closely linked to the overall livability and quality of life within the community. Additionally, the infrastructure conditions variable covered several fundamental aspects critical to the community environment, such as road quality, waste disposal systems, sewer availability, and waste management practices. By comprehensively accounting for these infrastructure elements, this system effectively represents the community environmental realities in China.

Our study identified infrastructure conditions and accessibility to public facilities as the 2 most critical CBE factors influencing the trajectory of depressive symptoms among middle-aged and older adults (Figure 2). First, these factors significantly predicted the initial level of depressive symptoms; middle-aged and older adults residing in communities with better infrastructure conditions and higher accessibility to public facilities exhibited significantly lower initial levels of depressive symptoms. This finding aligns with those reported in the existing literature. Fan et al [60] highlighted the crucial role of community factors such as infrastructure and senior centers in mitigating depressive symptoms among middle-aged and older adults. Similarly, Li et al [61] found that a lack of infrastructure in villages was positively associated with the prevalence of depression in older adults in rural China. Moreover, the availability of recreational facilities in urban communities was associated with a lower incidence of depressive symptoms [62]. Perceived proximity to community facilities is an important factor influencing depressive symptoms among low-income older adults. Improving accessibility to such facilities may help alleviate depressive symptoms [63]. From a mechanistic perspective, high-quality infrastructure provides a safer and more comfortable living environment and reduces the sources of psychological stress. Concurrently, convenient public facilities promote social participation and PA, offering environmental support for mental well-being.

Through a long-term analysis of depressive symptom trajectories, our study not only confirmed the effect of accessibility to public facilities on the initial level of depressive symptoms but also revealed its predictive role in the upward trajectory of depressive symptoms. Contrary to expectations, greater accessibility to public facilities was found to significantly accelerate the upward trajectory of depressive symptoms among middle-aged and older adults in China (Figure 2). This can be attributed to 2 reasons. First, higher accessibility may be associated with greater community activity and more complex social interactions, which can introduce additional environmental stressors, such as noise, social conflicts, or other pressures that negatively affect mental health [64]. Second, the inadequate service quality of public facilities and insufficient consideration of the needs of vulnerable populations could also play a key role. Middle-aged and older adults may have higher expectations of accessible public facilities; however, if these facilities fail to meet their needs—due to issues such as overcrowding, usage restrictions, or poor design—it may lead to disappointment or frustration [65]. In developing countries, public facilities are often designed with a focus on quantity and coverage, whereas service capacity and the needs of disadvantaged groups are frequently overlooked. This limitation may exacerbate mental health challenges in middle-aged and older adults by introducing potential risk factors.

Our study further demonstrates the significant effect of CBE on the initial level of total LTPA, emphasizing the importance of infrastructure conditions and accessibility to public transport. The results indicated that middle-aged and older adults living in communities with better infrastructure or higher public transport accessibility had significantly higher initial LTPA levels. These findings provide new insights into how the built environment influences health behaviors among middle-aged and older adults. Additionally, middle-aged and older adults in communities with better infrastructure and higher public facility accessibility engaged in significantly lower initial OPA. Furthermore, infrastructure conditions positively predicted a declining trajectory in OPA levels over time (Figure 2). As residential communities are not direct venues for occupational activity, a plausible explanation is that infrastructure conditions and public facility accessibility reflect the overall quality, location advantages, and amenities provided within the community. In China, residents of higher-tier communities are typically part of the nonlabor force population, while manual laborers tend to reside in communities with poorer infrastructure and fewer amenities [66]. This community-level differentiation significantly impacts the intensity and type of occupational activity, reflecting the observed community segregation and spatial stratification within Chinese society [67]. However, due to the lack of data on community-level economic variables, this study could not fully account for the potential influence of these factors on the results. Further research is needed to validate these associations and explore the underlying mechanisms.

### Implications

Our findings could offer valuable insights for public health management practices and community environmental planning, with relevance to other developing countries or regions. When formulating community PA guidelines, it is crucial to consider the distinct effects of different types of PA. LTPA should be integrated into community mental health intervention programs, with a focus on encouraging middle-aged and older adults to engage in activities such as walking. However, relevant authorities should also guide middle-aged and older adults in reducing the intensity of OPA, optimizing workplace conditions and workflows, and providing mental health support. This recommendation is particularly critical for developing countries, where physical labor constitutes a significant portion of the workforce.

From the perspective of CBE, improving community infrastructure and enhancing the accessibility of public facilities are key strategies for preventing and addressing mental health issues in middle-aged and older adults, while also promoting PA. This includes, but is not limited to, upgrading road quality, improving water and sewage systems, and enhancing electricity services. These measures can lead to better depressive symptom outcomes and encourage LTPA in middle-aged and older populations. Furthermore, improving public transportation accessibility has the potential to promote LTPA in this demographic. However, we recommend that governments carefully consider the potential environmental stressors associated with enhanced public facilities and focus on improving their service capacity and quality. Special attention should be given to addressing the needs of vulnerable populations to mitigate negative effects and more effectively promote community mental health.

### Limitations and Future Research

First, we excluded participants with missing PA or depressive symptom scores. While this approach is common, it may introduce bias, as it is challenging to determine whether the missing values are associated with participants who have lower PA levels or higher depressive symptom scores. Second, although the findings offer critical empirical support for the PA paradox, the study is constrained by its design and data availability, with limited consideration of factors such as workplace environment, working conditions, and job autonomy. These factors may mediate or confound the relationship between PA and mental health. Future research should incorporate these variables to comprehensively elucidate the complex mechanisms linking OPA and mental health. Third, the wave 2020 data used in this study were collected during the COVID-19 pandemic, which may have influenced the results. Fourth, due to space constraints and the study’s objectives, insufficient attention was given to subgroup analyses or population heterogeneity, which future research could explore in greater detail. Finally, the CBE data in the CHARLS were published for only 1 wave without subsequent follow-up, limiting this study’s ability to examine changes in the role of CBE in the relationship between PA and depressive symptom trajectories. Future research should incorporate dynamic geographical information to capture the effects of CBE changes on mental health or PA trajectories.

### Conclusions

Communities play an integral role in the daily lives of residents. This study offers valuable insights from developing countries by highlighting the effects of PA on depressive symptom trajectories and the role of CBE factors as physical determinants. The findings reveal that the impact of PA on depressive symptoms varies by domain and intensity. Overall, higher levels of LTPA were associated with lower depressive symptom levels among middle-aged and older adults, whereas OPA, particularly at moderate-to-vigorous intensities, had negative effects on depressive symptoms. Additionally, OPA exhibits a dual effect: while a higher initial level of total OPA was associated with a higher initial level of depressive symptoms, it also contributed to slowing the upward trajectory of depressive symptoms over time. Among the CBE factors, infrastructure conditions and accessibility to public facilities emerged as the most critical determinants influencing depressive symptom trajectories. Better infrastructure and greater accessibility to public facilities were linked to lower initial levels of depressive symptoms; however, greater accessibility to public facilities might exacerbate the upward trajectory of depressive symptoms. Furthermore, infrastructure conditions and accessibility to public transport played positive roles in promoting LTPA.

These findings provide novel perspectives on the PA paradox in mental health research, offering valuable guidance for developing more precise public health strategies. Such strategies should aim to promote PA types that effectively reduce depressive symptoms while minimizing potential adverse effects. By identifying the specific impacts of CBE factors, this study underscores that optimizing the built environment is not merely a physical enhancement but also a vital public health approach to improving residents’ mental health. We advocate for enhancing community environments to encourage beneficial PA and alleviate depressive symptoms among middle-aged and older adults.

## Appendix

Multimedia Appendix 1 Additional analyses.

## Abbreviations

CBE: community-built environment
CES⁃D: Center for Epidemiologic Studies Depression Scale
CHARLS: China Health and Retirement Longitudinal Survey
LGCM: latent growth curve modeling
LRT: likelihood ratio test
LTPA: leisure-time physical activity
MET: metabolic equivalent
OPA: occupational physical activity
PA: physical activity
RCI: Reliable Change Index
WHO: World Health Organization
