# Supplementary material for: Identifying Community-Built Environment’s Effect on Physical Activity and Depressive Symptoms Trajectories Among Middle-aged and Older Adults: Chinese National Longitudinal Study
Source: JMIR Public Health Surveill. 2025 Jan 13;11:e64564. doi: 10.2196/64564 (PMC11773281; doi:10.2196/64564)
Supplement: Multimedia Appendix 1 [file publichealth_v11i1e64564_app1.docx]

**Appendix A**

**(1) Min-Max Normalization method**

$X_{norm}=\frac{X - X_{min}}{X_{max} - X_{min}}$ (1)

Where *X_norm_* represents the normalized value after scaling, *X* is the original data value to be normalized, *X_min_* is the minimum value of the variable, *X_max_* is the maximum value of the variable.

**(2) Further introduction of China Health and Retirement Longitudinal Survey**

The sampling design of the China Health and Retirement Longitudinal Study (CHARLS) aimed to ensure unbiasedness and representativeness of the sample. A four-stage sampling process was implemented at the county (district), village (community), household, and individual levels.

Specifically, at the county and village levels, CHARLS adopted a probability proportional to size (PPS) sampling method based on population size. In the county-level sampling stage, 150 counties or districts were randomly selected from 30 provincial-level administrative units in China (excluding Tibet, Taiwan, Hong Kong, and Macau) using the PPS method. This selection was stratified by region, urban-rural classification, and GDP, and based on 2009 population data. In the village-level sampling stage, three villages or communities were randomly selected from each of the 150 counties or districts using the PPS method, based on the 2009 resident population in each village or community. This process resulted in the selection of 450 villages or communities. To minimize discrepancies in population information, the 2009 resident population data for the 450 village-level units were cross-checked with 2007 data during sampling. For villages or communities with significant differences between the two datasets, verification was conducted with the National Bureau of Statistics. Additionally, selected villages or communities were validated through official communication by the Chinese Center for Disease Control and Prevention, further ensuring sampling quality. Following the completion of village/community sampling, a specialized mapping software system, CHARLS-GIS, was developed and deployed for field mapping and the collection of household information to create an accurate household sampling frame.

The Biomedical Ethics Review Committee of Peking University (IRB00001052-11015) reviewed and approved the data collection, ensuring the study protocol adhered to the ethical standards of the 1975 Declaration of Helsinki. All participants gave informed consent after being thoroughly informed about the study.

**(3) Definitions and data collection methods for different types of physical activity in the China Health and Retirement Longitudinal Study questionnaire**

Ask DA051 for each type of physical activity (PA), including:

1、Vigorous-intensity activity (Vigorous activities can cause shortness of breath. Exam ples of vigorous-intensity activities include carrying heavy stuff, digging, hoeing, aerobic workout, bicycling at a fast speed, riding a cargo bike/motorcycle, etc.)

2、Moderate activity (Moderate activities can make you breathe faster than usual. Ex amples of moderate activities include carrying light stuff, bicycling at a normal speed, mopping, Tai-Chi, and speed walking. )

3、Mild activities such as walking (walking from one place to another place at a work place or home, and taking a walk for leisure, sports, exercise or entertainment)

If DA051 = 1, ask DA052 to DA051_1 for each type of physical activity.

(DA051) Please recall the [preload the type of physical activity] that you have taken part in for at least 10 minutes every time in a week. Do you usually take this type of activity for at least 10 minutes every week?

1. Yes

2. No

(DA052) How many days a week do you take part in [preload the type of physical activity] for at least 10 minutes?

(DA053) During the days you take part in [preload the type of physical activity], how long do you do [preload the type of physical activity] every day?

1. < 2 hours

2. ≥ 2 hours **→** Skip to DA055

(DA054) During the days you take part in [preload the type of physical activity], how long do you do [preload the type of physical activity] every day?

1. < 30 minutes → Skip to DA051_1

2. ≥ 30 minutes → Skip to DA051_1

(DA055) During the days you take part in [preload the type of physical activity], how long do you do [preload the type of physical activity] every day?

1. < 4 hours

2. ≥ 4 hours

(DA051_1) What’s the purpose for doing these physical activities, for entertainment, job demand or exercise in doing these physical activities?

1. Job demands

2. Entertainments

3. Exercise

4. Other

**(4) Measurement of Physical Activity Metabolic Equivalent (MET)**

**1. Calculation Method**
The MET for physical activity was calculated based on each participant’s self-reported weekly PA intensity (vigorous, moderate, and walking), duration (more than 4 hours, 2–4 hours, 30 minutes–2 hours, and less than 30 minutes), and frequency (1–7 days per week). The total MET for PA was obtained by summing the METs for each activity type, calculated as follows:

Walking MET-minutes/week = 3.3*walking minutes*walking days

Moderate MET-minutes/week = 4.0*moderate-intensity activity minutes*moderate days

Vigorous MET-minutes/week = 8.0*vigorous-intensity activity minutes*vigorous-intensity days

Total PA MET-minutes/week = sum of Walking + Moderate + Vigorous MET-minutes/week scores

For each activity type, the MET coefficients were derived from the International Physical Activity Questionnaire (IPAQ): walking = 3.3, moderate PA = 4.0, and vigorous PA = 8.0 [1].

2.**Data cleaning and elimination of outliers**
First, the cumulative daily duration of each activity was converted into minutes. Data with missing frequency or duration values were excluded from the analysis. Participants whose total cumulative daily duration of the three PA intensities exceeded 960 minutes (16 hours) were considered outliers and excluded from the analysis[2].

3.**Data Truncation**
We used the median value of each duration option to represent the duration of each PA intensity. Considering that a minimum continuous duration of 10 minutes is required for health benefits from PA, activities lasting less than 10 minutes per session were excluded [3]. Additionally, if the reported daily duration of any PA intensity exceeded 4 hours, it was capped at 240 minutes.

**References**

1. R. C. IPAQ. Guidelines for data processing and analysis of the International Physical Activity Questionnaire (IPAQ)-short and long forms. http://www. ipaq. ki. se/scoring. pdf, 2005.

2. M. Fan, J. Lyu, P. He. [Chinese guidelines for data processing and analysis concerning the International Physical Activity Questionnaire]. Zhonghua liu xing bing xue za zhi = Zhonghua liuxingbingxue zazhi, 2014, 35(8): 961-964.

3. X. Li, W. Zhang, W. Zhang, et al. Level of physical activity among middle-aged and older Chinese people: evidence from the China health and retirement longitudinal study. BMC Public Health, 2020, 20(1): 1682.

**Appendix B**


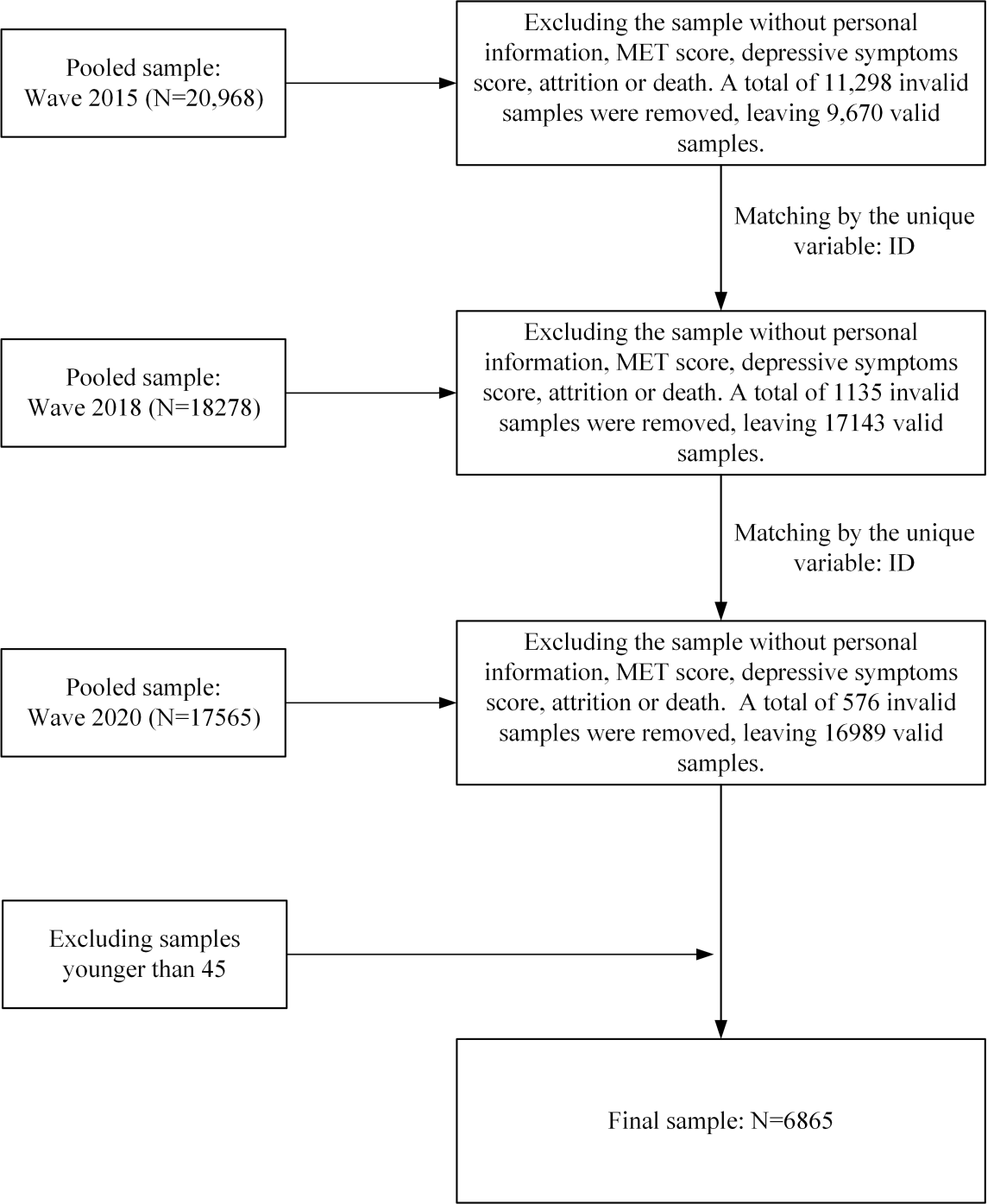


**Fig. S1. Flow of the sample screening**

**Table S1.** Community built environment variable system constructed from the community questionnaire of China Health and Retirement Longitudinal Survey

| **Built environment element** | **Variable** | **Calculation method** | **Calculation content** | **Question number** |
| --- | --- | --- | --- | --- |
| Density | Residential density | Residential density | Community population/total area of the community | JA003, JC001 |
|  | Density of public facilities | Total Number of basic public facilities in the village or community/total area of the community | A total 12 of basic public facilities are included in the calculation: kindergartens, primary schools, junior middle schools, senior high schools, post offices, libraries, police stations, banks, theatres, nursing homes, convenience stores, farmers' markets, supermarkets. | JA003, JB028 |
| Diversity | Diversity of sports venues | Diversity of community sports venues in your village | A total 8 of sports venues are included in the calculation: basketball court, swimming pool, outside exercising facilities, table tennis court, room for card games and chess games, room for Ping Pong, dancing team or other exercise organizations, entertainment facilities. The diversity level was calculated as described above. | JB029 |
|  | Diversity of senior care facilities | Diversity of activities venues for the senior | A total 7 of senior care facilities are included in the calculation: activity center for the senior, senior association, nursing home, senior care center, association for calligraphy and painting, organization for helping the senior and the handicapped, home-based senior care service centers. The diversity level was calculated as described above. | JB029 JB030 JB037 |
| Destination accessibility | Accessibility of public facilities | Average distance from the community office to the most commonly used this type of facility | Actual distance of each of the 12 basic public facilities (answer 0 if this facility is located in the village/community) (km). | JB028 |
| Distance to transit | Accessibility of public transport | How many bus lines are accessible in this community | Actual lines. | JB003 |
|  |  | Actual distance from the community office to the most commonly used bus stop | Actual distance. | JB004 |
| Infrastructure | Infrastructure conditions | The main type of road in the community | A total of 3 types, where (1) pathway/dirt/unpaved road=1, (2) sand-stone road=2, (3) paved road =3. | JB001 |
|  |  | Number of days when roads are impassable | Actual days. | JB002 |
|  |  | The proportion of households using purified tap water | Number of users using purified tap water/total number of households. | JB006 |
|  |  | Whether there is a sewer system | Yes=1 point, No=0 point. | JB010 |
|  |  | Disposal of waste | A total of 5 types of waste disposal, where (1) moved away by truck =5 points, (2) buried in this village =4 points, (3) burn away =3 points, (4) put into nearby river =2 points, (5) do not manage =1point. | JB012 |
|  |  | The proportion of households using electricity | Number of users using purified tap water / total number of households. | JB013 |
|  |  | The main type of toilet in the village/community | A total of 5 types, where (1) inside toilet with water =5, (2) inside toilet without water =4, (3) outside toilet with water =3, (4) outside public toilet without water =2, (5) open-air =1 | JB017 |

**Table S2.** Descriptive statistical characteristics of the study sample

| **Variable** | **Definition** | **Mean (SD)/N (%)** |
| --- | --- | --- |
| **Demographic and socioeconomic status** |  |  |
| Age | Range = 45-105 | 58.98 (8.96) |
| Sex |  |  |
| Male | Male = 0 | 3219 (46.89%) |
| Female |  | 3646 (53.11%) |
| Education level |  |  |
| Illiteracy | Illiteracy = 0 | 1571 (22.88%) |
| Primary and lower |  | 3018 (43.96%) |
| Middle, high, and vocational school |  | 2167 (31.57%) |
| Three-year college/bachelor’s degree and higher |  | 109 (1.59%) |
| Cohabit status |  |  |
| With cohabiting partner | With cohabiting partner = 0 | 5767 (84.01%) |
| Without cohabiting partner |  | 1098 (15.99%) |
| Marital status |  |  |
| Married | Married = 0 | 6836 (99.58%) |
| Other |  | 29 (0.42%) |
| Personal annual income  (Chinese yuan) |  | 15712.63 (20049.21) |
| Smoking |  |  |
| Still smoke | Still smoke = 0 | 1879 (27.37%) |
| Do not smoke |  | 4986 (72.63%) |
| Drinking |  |  |
| Drinking more than once a month | Drink more than once a month = 0 | 1861 (27.11%) |
| Drinking less than once a month |  | 604 (8.80%) |
| Do not drink |  | 4400 (64.09%) |
| Disability |  |  |
| Disabled | One or more physical/brain/damage/vision/hearing/speech disability | 872 (12.70%) |
| Non-disabled |  | 5993 (87.30%) |
| Number of chronic diseases | Diagnosed with any of the following: Hypertension, dyslipidemia, diabetes or high blood sugar, cancer or malignant tumor, chronic lung diseases, liver disease, heart attack, stroke, kidney disease, stomach or other digestive disease, emotional, nervous, or psychiatric problems, memory-related disease, arthritis or rheumatism, asthma. Yes = 1, No = 0, calculate the total number of chronic diseases, range = 0-10. | 2.10 (1.01) |
| Score of housing characteristic (range = 0-11) | presence of: elevator, barrier-free facilities, toilet flushing, electricity, running water, bathing facilities, gas and natural gas, heating, broadband, air purifier, tidiness. Yes = 1, No = 0, calculate the total score of housing quality for each respondent, range = 0-11. | 4.06 (1.72) |
| **Community type** |  |  |
| Urban community |  | 1127 (16.42%) |
| Urban-Rural Integration Zone |  | 475 (6.92%) |
| Rural community |  | 5241 (76.34%) |
| Special Area |  | 22 (0.32%) |
| **Community built environment** |  |  |
| Traffic and accessibility | range = 0-1 | 0.73 (0.17) |
| Infrastructure conditions | range = 0-1 | 0.50 (0.37) |
| Basic public facilities | range = 0-1 | 0.47 (0.14) |
| Sports venue | range = 0-1 | 0.27 (0.28) |
| senior care facility | range = 0-1 | 0.21 (0.27) |
| Medical facility | range = 0-1 | 0.38 (0.10) |

**Table S3.** Reliability change index statistics for depressive symptoms scores across waves

| **Category** | **2015-2018** | **2018-2020** | **2015-2020** |
| --- | --- | --- | --- |
| Total sample (N) | 6865 | 6865 | 6865 |
| Significant increase (RCI > 1.96) | 805 (11.73%) | 766 (11.16%) | 948 (13.81%) |
| Significant decrease (RCI < -1.96) | 554 (8.07%) | 616 (8.97%) | 526 (7.66%) |
| No significant change  (-1.96 ≤ RCI ≤ 1.96) | 5506 (80.20%) | 5483 (79.87%) | 5391 (78.53%) |
| Abbreviations: RCI, reliability change index. | | | |

**Table S4.** Fit index of conditional latent growth curve modeling for physical activity

| Path | Chi-Square (degrees of freedom) | RMSEA (90% CI) | CFI (TLI) | SRMR |
| --- | --- | --- | --- | --- |
| Total LTPA → depressive symptoms | 12.263 (7) | 0.010 (0.000, 0.020) | 0.999 (0.995) | 0.007 |
| Low intensity LTPA → depressive symptoms | 12.486 (7) | 0.011 (0.000, 0.020) | 0.999 (0.994) | 0.008 |
| Moderate-to-vigorous intensity LTPA → depressive symptoms | 9.668 (7) | 0.007 (0.000, 0.018) | 1.000 (0.997) | 0.005 |
| Total OPA → depressive symptoms | 28.113 (7) | 0.021 (0.013, 0.029) | 0.997 (0.979) | 0.010 |
| Low intensity OPA → depressive symptoms | 13.089 (7) | 0.011 (0.000, 0.021) | 0.999 (0.994) | 0.007 |
| Moderate-to-vigorous intensity OPA → depressive symptoms | 26.606 (7) | 0.020 (0.012, 0.029) | 0.997 (0.980) | 0.010 |
| Parallel process LGCM | 46.860 (41) | 0.005 (0.000, 0.010) | 0.999 (0.998) | 0.006 |
| Abbreviations: RMSEA, root mean square error of approximation; CI, confidence interval; SRMR, standardized root mean square residual; TLI, Tucker-Lewis index; CFI, comparative fit index; LTPA, leisure-time physical activity; OPA, occupational physical activity; LGCM, latent growth curve modeling.  Note: *P < .05; **P < .01; ***P < .001. | | | | |
